# Solitary Intra-Osseous Myofibroma of the Jaw: A Case Report and Review of Literature

**DOI:** 10.3390/children4100091

**Published:** 2017-10-24

**Authors:** Anita Dhupar, Karla Carvalho, Poonam Sawant, Anita Spadigam, Shaheen Syed

**Affiliations:** Department of Oral & Maxillofacial Pathology, Goa Dental College & Hospital, Goa 403202, India; dranitadhupar@gmail.com (A.D.); p_sawant2010@yahoo.in (P.S.); anita.spadigam@gmail.com (A.S.); drshaheen73@gmail.com (S.S.)

**Keywords:** solitary myofibroma, intra-osseous, mandible

## Abstract

Myofibroma is a rare benign spindle cell neoplasm in children that usually affects both soft tissue and bone in the head and neck region. Approximately one third of these cases are seen within jaw bones as solitary lesions. Solitary intra-osseous myofibroma of the jaw bone shares its clinical, radiographic and histological features with other spindle cell tumors. The rarity of this lesion can make diagnosis difficult for clinicians and pathologists. We report a case of a solitary intra-osseous myofibroma in the mandible of a nine-year-old child.

## 1. Introduction

Myofibroma is a rare benign lesion of myoid contractile spindle cells, affecting both soft tissues and bone in children. It has two distinct clinical presentations: solitary and multicentric [1,2,3]. Solitary myofibroma in the oral and maxillofacial region is uncommon (accounts for 2% of all cases) and usually affects the oral and peri-oral soft tissues [4]. Intra-osseous myofibroma (IM) of the jaw bones is sporadic and shows a predilection for the lower jaw [3,5]. Due to its rare nature, combined with the nonspecific clinical, radiographic and histologic features, this lesion runs the risk of being misdiagnosed as a malignant and/or aggressive spindle cell neoplasm [1,2,6,7].

To the best of our knowledge, 40 cases of solitary myofibroma of the mandible in the pediatric age group have been reported in literature [8,9]. We report a case of solitary intra-osseous myofibroma in the lower jaw of a nine-year-old child and present a review of literature of the same lesion in the jaw bones.

## 2. Patient Description

A previously healthy nine-year-old girl presented to the pediatric department of her local dental hospital with a complaint of a swelling of the left side of her lower jaw. The parents of the child had noticed that the swelling had gradually increased in size over the last four months. The child complained of a dull pain which was continuous and localized to the left side of her lower jaw.

Neither the parents nor the child reported any trauma to the area in the recent past. No history or evidence of infection and/or inflammation induced symptoms such as fever, paresthesia or pus-discharge from the affected region were noted. However, the parents mentioned that the child had undergone an extraction of her left mandibular second deciduous molar, four months previously, due to severe caries. The parents reported that the extraction had been uneventful.

**Figure 1:**
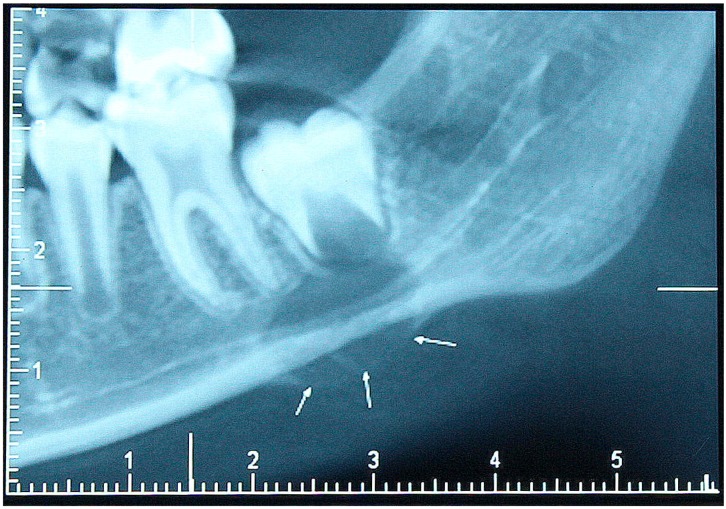
Cone beam computed tomographic image shows a well-defined radiolucent lesion around the root of developing second molar and concomitant expansion of the facial cortical bone.

The swelling was located at the left angle of the lower jaw, had a bony hard consistency and was tender. It measured approximately 2 cm × 2 cm in dimension and resulted in a mild asymmetry of the face. Intra-oral examination revealed an expansion of the jaw bone, which on the facial aspect had resulted in obliteration of the vestibule in the affected area. A cone beam computed tomography (CBCT) investigation showed a well-defined radiolucent area surrounding the forming root of the erupting lower left second molar. The inferior alveolar canal appeared uninvolved although localized destruction of the facial bone plate was evident (Figure 1).

The complete blood count of the child was unremarkable. All parameters—namely, white blood cell count, red blood cell count, platelet count, hemoglobin and hematocrit—were within normal limits. Based on the clinical and radiographic data, a provisional diagnosis of an odontogenic cyst/tumor was made.

**Figure 2:**
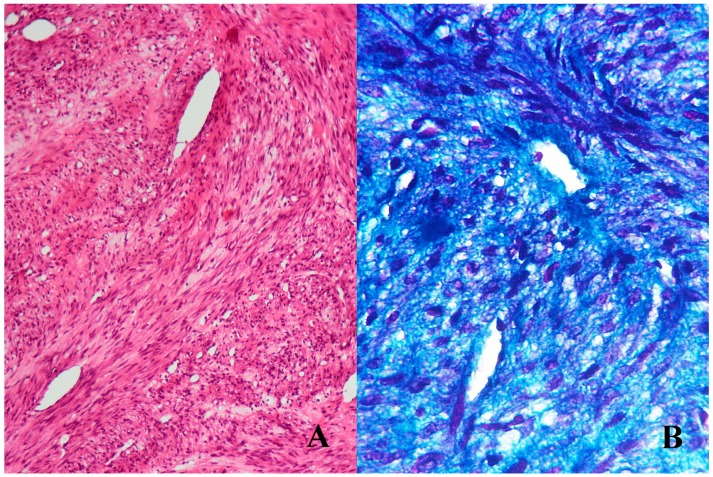
(**A**) (100×) Hematoxylin and eosin stained section shows fascicular and cellular areas characterized by polygonal cells at the center and elongated cells at the periphery. (**B**) (400×) Masson’s trichrome stain highlights the highly fibrous stroma.

An incisional biopsy was advised, and revealed interlacing fascicles of spindle shaped cells arranged in a bi-phasic pattern with cleft-like and branching vascular spaces. Characteristic zoning phenomenon was exhibited by peripheral spindle cells with oval to tapering nuclei, whereas the central round to polygonal cells had scant cytoplasm and hyperchromatic nuclei. Abnormal mitotic figures and areas of necrosis were absent. A special stain technique for collagen using Masson’s trichrome stain was used to quantify the stromal component within the lesional mass. The special stain demonstrated an excess of collagen (blue) around the cellular component (pink) (Figure 2).

**Figure 3:**
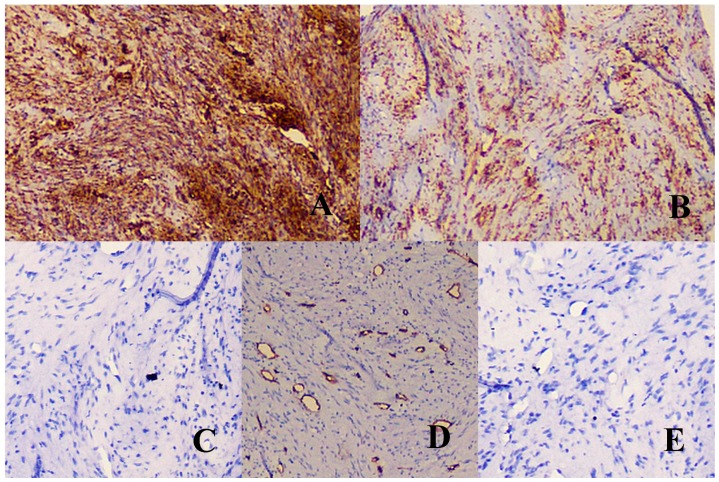
Immunohistochemical marker panel. (**A**,**B**) Tumor cells are positive for vimentin and alpha-smooth muscle actin (α-SMA) markers, respectively. (**C**,**E**) Tumor cells are negative for S-100 and desmin markers respectively. (**D**) CD-34 marker is positive in the blood vessels but negative in the tumor cells.

In order to confirm the spindle cell origin, an immunohistochemical marker panel was run comprising of the following markers: CD-34, vimentin, desmin, S-100, alpha-smooth muscle actin (α-SMA). With the exception of vimentin and α-SMA, the spindle cell tumor was negative for all the other markers (Figure 3). Collating the findings, the investigators arrived at a diagnosis of an intra-osseous myofibroma.

The child was then referred to a pediatric surgical unit, specializing in head and neck pathology where enucleation and curettage of the lesion was performed. The tooth associated with the lesion was retained. On communicating with the surgical unit, we were happy to learn that the patient is asymptomatic, one year after surgery.

## 3. Discussion

Smith, in 1989, coined the term myofibromain order to differentiate the solitary form from multicentric myofibromatosis [10]. This benign neoplasm is made up of cells thought to possess myofibroblastic phenotype (i.e., fibroblastic and smooth muscle) [11]. Currently, however, it is classified as a perivascular tumor by the World Health Organization 2013 revised classification of tumors of soft tissues and bone [12].

A number of hypotheses have been put forth to explain the etiopathogenesis of myofibroma. Myofibroblasts are thought to aid in wound healing. An injury or trauma to the affected area therefore is hypothesized to result in myofibroblastic proliferation in that region [13]. Intra-uterine exposure to estrogen can also result in the development of such lesions in children. Studies have reported estrogen-induced lesions with similar histologic features to IM in guinea pigs [14]. Other authors suggest that genetic aberrations with an autosomal or recessive mode of inheritance could contribute to the genesis of this tumor [15,16,17]. One study claimed that primitive vascular progenitor cells have a role to play in the development of myofibroma and considered it to be a reactive vascular lesion [18].

Solitary myofibroma is seen most commonly in the first decade of life and no gender predilection has been observed [2]. In children, it commonly presents as an intra-osseous mass affecting the cranio-facial skeleton. It shows predilection for the lower jaw, as is seen in this particular case. Posterior regions of the lower jaw are affected more often than the anterior region [2,4,7]. Thus far in the literature, only two cases of IM involving the upper jaw have been documented [2]. Soft tissue myofibroma has been reported in oral and peri-oral soft tissues such as tongue, lips, palate and floor of the mouth [19].

Intra-osseous myofibroma usually presents as an asymptomatic swelling with some reports mentioning asymmetry of the face caused by the lesion, as was seen in this case. Rare reports of IM causing restricted mouth opening and mental nerve paresthesia have been documented in literature [2,20].

This case exhibited the most common radiographic feature seen in IM: unilocular radiolucency with bone expansion. Other possible radiographic presentations include bilocular and multilocular radiolucencies, at times showing indistinct margins, perforation of bone or marginal sclerosis [2,21]. Odontogenic tumors, such as unicystic ameloblastoma, odontogenic keratocyst and central odontogenic fibroma are the most probable radiographic differential diagnosis for unilocular IM of the jaw [9,22]. Solid multicystic ameloblastoma, aneurysmal bone cyst and central hemangioma could be considered differential diagnoses for a multilocular IM of the jaw [9,23].

Myofibroma, both arising in soft tissue and bone shows a characteristic histopathological bi-phasic pattern composed of elongated spindle cells at the periphery and polygonal cells with hyperchromatic nuclei at the center [4,20,24]. This microscopic picture has to be differentiated from both benign spindle cell tumors such as leiomyoma, schwannoma, neurofibroma, solitary fibrous tumor, desmoplastic fibroma, inflammatory myofibroblastic tumor, nodular fasciitis, benign fibrous histiocytoma, desmoid tumor, myopericytoma and malignant spindle cell neoplasms such as low grade fibrosarcoma, leiomyosarcoma myofibrosarcoma and rhabdomyosarcoma [4,7,9,24,25,26,27,28,29,30,31,32,33,34,35,36,37,38,39,40,41,42]. Immunohistochemical markers should be used to differentiate spindle cell tumors with similar histopathologic features to confirm the cell of origin [4,9,20,21,24]. Characteristically, the cellular component of IM shows positivity for α-SMA, vimentin and negativity for other muscle, neural and vascular markers, thus confirming the myofibroblastic proliferation [2] (Table 1).

The well-circumscribed benign nature and low recurrence rate of IM makes conservative surgery the treatment of choice [2,4,23,43]. Enucleation and curettage are the techniques adopted for most cases of IM, as was done in this case [2,23]. Recurrence seems to be related to poor surgical approach, which could arise due to anatomic constraints in jaw bones. Deciduous and permanent tooth buds in relation to the lesion can be retained without them contributing to a recurrence of the lesion, as was done in this case [44]. Although radical treatment measures such as mandibular segmental resection followed by rehabilitation using reconstruction surgical plates and bone grafts have been mentioned in the literature, the low recurrence rate seems to support a more conservative approach. Aggressive surgery would be warranted for extensive lesions where conservative surgical methods might result in the incomplete removal of the tumor. After a thorough review of literature, it is evident that no specific post-surgical follow-up regime exists. [2,4,43].

Intra-osseous myofibroma requires an exhaustive clinical-histological-radiological work-up combined with an efficient immunohistochemical marker panel helps to facilitate its diagnosis.

## Figures and Tables

**Table 1:** Microscopic features and immunohistochemical markers that differentiate intra-osseous myofibroma from other benign and malignant spindle cell tumors that can involve the jaw bone in children [2,4,7,9,20,21,24,25,26,27,28,29,30,31,32,33,34,35,36,37,38,39,40,41,42].

Pathology	Microscopic Features	Immunohistochemistry
Benign Tumors		
Intra-osseous myofibroma	Alternating growth pattern show fascicular and cellular areas characterized by polygonal cells at the center and elongated cells at the periphery. Absence of pleomorphism, nuclear atypia and mitosis.	α-SMA and vimentin positive.
Leiomyoma	Homogenous fascicular pattern made up of spindle cells with cigar shaped nucleus and bright eosinophilic cytoplasm.	Desmin, α-SMA, muscle specific actin (HHF-35), calponin positive.
Schwannoma	Cellular areas showing palisading growth pattern (Antoni A) intermixed with fibrillar unorganized cellular areas (Antoni B).	Diffuse S-100 podoplanin, calretinin, neurofibromin, CD34, glial fibrillary acidic protein (GFAP), collagen IV positive. Occasionally positive for cytokeratin (CK).
Neurofibroma	Spindle shaped cells with wavy nuclei showing fascicular or storiform growth pattern, at times myxoid areas are seen.	Expression of markers in descending order of immuno-reactivity S-100, CD34, SRY (sex determining region Y)-box 10 (Sox 10), collagen IV, calretinin, podoplanin, epithelial membrane antigen (EMA) positive.
Solitary fibrous tumor	No distinct cellular pattern of growth, however staghorn branching of blood vessels and perivascular hyalinization is seen.	CD-34, signal transducer and activator of transcription-6 (STAT-6), CD-99, B-cell CLL/lymphoma 2 (Bcl-2) (>85%) positive. Nuclear reactivity for β-catenin (22–67%).
Desmoplastic fibroma	Monomorphic fascicles of spindle cells admixed with abundant wavy collagen fibers. Absence of branching vasculature.	Vimentin (92%), β-catenin (50%) and occasional positivity for α-SMA.
Inflammatory myofibroblastic tumor	Three patterns of plump spindle cell arrangements have been described, along with an infiltrate of chronic inflammatory cells: spindle cells within a myxoid stroma, fascicular or storiform arrangement in a collagenous stroma and hypocellular elongated spindle cell component in a dense collagenous stroma.	Positive markers include: α-SMA, HHF-35, desmin (~50%), anaplastic lymphoma kinase protein (ALK-1) (30–60%), both anti-pan cytokeratin (AE1/AE3) and anticytokeratin for cytokeratin peptide 8 (CAM 5.2) (<35%) respectively.
Nodular fasciitis	Three patterns have been described: Type 1: Myxomatous vascular and acellular central stroma with plump fibroblasts at the periphery. Type 2: Cellular stroma with slit like vascular spaces. Type 3: Fibromatous stroma which contains interlacing fiber bundles, spindle shaped fibroblasts and capillaries.	Vimentin, HHF-35, α-SMA positive.
Benign fibrous histiocytoma	Fibroblast and histiocyte proliferation in a storiform pattern, with occasional multinucleated giant cells and foam cells.	Vimentin, CD-68, 1-antitrypsin, 1-antichymotrypsin positive.
Desmoid tumor	Mature spindle shaped cell proliferation separated by bundles of fibrous tissue.	Focal positivity for α-SMA, desmin and nuclear reactivity for β-catenin.
Myopericytoma	Oval or spindle shaped cells with a concentric peri-vascular arrangement.	α-SMA, smooth muscle myosin heavy chain, h-caldesmon, and calponin positive.
**Malignant Tumors**		
Low-grade fibrosarcoma	Malignant fibroblasts show typical herring bone pattern, combined with high mitotic activity and nuclear atypia.	Vimentin positive. Variable positive expression of: α-SMA, HHF35, neuron-specific enolase, desmin, S-100, CD34, and CK.
Leiomyosarcoma	Palisaded pattern with densely packed spindle cells with fibrillar eosinophilic cytoplasm showing indistinct cell borders. Pleomorphism and high mitotic index is evident.	Diagnosis is based expression of any two of the following markers: α-SMA, desmin, HHF35, calponin. EMA and CK are occasionally positive (10–30%).
Myofibrosarcoma	Show varied patterns which include fibrosarcoma like areas. Nuclear pleomorphism is ubiquitous.	Vimentin (100%), Fibronectin (100%), α-SMA (~90%), HHF35 (~78%), calponin (67%), and desmin (~20%) positive.
Rhabdomyosarcoma	Ovoid or elongated rhabdomyoblasts with eosinophilic granular cytoplasm. Few blast cells show cross striations. Multinucleated tumor giant cells and abnormal mitotic figures are occasionally seen in a myxoid stroma.	Nuclear reactivity for myogenin and myogenic differentiation 1 (MyoD1). Diffuse desmin and rare α-SMA positive (~10%).
